# A Novel Characterization and Performance Measurement of Memristor Devices for Synaptic Emulators in Advanced Neuro-Computing

**DOI:** 10.3390/mi11010089

**Published:** 2020-01-13

**Authors:** AlaaDdin Al-Shidaifat, Shubhro Chakrabartty, Sandeep Kumar, Suvojit Acharjee, Hanjung Song

**Affiliations:** 1 Department of Nanoscience and Engineering, Centre for Nano Manufacturing, Inje university, Gimhae 50834, Korea; alaaddinsh@hotmail.com; 2 Department of Electronics and Communication Engineering, National Institute of Technology Karnataka, Surathkal, Mangaluru 575025, Karnataka, India; fedrer.engg@gmail.com; 3 Department of Electronics and Communication Engineering, National Institute of Technology Agartala, Jirania 799046, Tripura, India; acharjeesuvo@gmail.com

**Keywords:** neuro-computing, nanoparticles, synaptic and neurons, titanium dioxide (TiO_2_)

## Abstract

The advanced neuro-computing field requires new memristor devices with great potential as synaptic emulators between pre- and postsynaptic neurons. This paper presents memristor devices with TiO_2_ Nanoparticles (NPs)/Ag(Silver) and Titanium Dioxide (TiO_2_) Nanoparticles (NPs)/Au(Gold) electrodes for synaptic emulators in an advanced neurocomputing application. A comparative study between Ag(Silver)- and Au(Gold)-based memristor devices is presented where the Ag electrode provides the improved performance, as compared to the Au electrode. Device characterization is observed by the Scanning Electron Microscope (SEM) image, which displays the grown electrode, while the morphology of nanoparticles (NPs) is verified by Atomic Force Microscopy (AFM). The resistive switching (RS) phenomena observed in Ag/TiO_2_ and Au/TiO_2_ shows the sweeping mechanism for low resistance and high resistance states. The resistive switching time of Au/TiO_2_ NPs and Ag/TiO_2_ NPs is calculated, while the theoretical validation of the memory window demonstrates memristor behavior as a synaptic emulator. Measurement of the capacitor–voltage curve shows that the memristor with Ag contact is a good candidate for charge storage as compared to Au. The classification of 3 × 3 pixel black/white image is demonstrated by the 3 × 3 cross bar memristor with pre- and post-neuron system. The proposed memristor devices with the Ag electrode demonstrate the adequate performance compared to the Au electrode, and may present noteworthy advantages in the field of neuromorphic computing.

## 1. Introduction

The memristor has an inherent memory, and has for the decades shown significant steps for developing in an advanced neurocomputing paradigm. The advanced system enables memristor devices as synaptic emulators for data processing between presynaptic and postsynaptic neurons. An artificial neuron cell communicates electrically with the other cell, through synapses [1,2]. The strength of that transmitted signal relates to synaptic weight. McCulloch and Pitts proposed the first time-independent neuron model in the year 1943 [3]. They manifested their work by showing the complex pattern of the brain by connecting basic cells. The basic cells are known as neurons. In the year 1952, Hodgkin and Huxley [4] implemented the electronic circuit by showing the electrical properties of neurons. From the last couple of decades, a number of researchers have worked phenomenally for mimicking the neurons by creating an artificial paradigm. 

**Figure 1:**
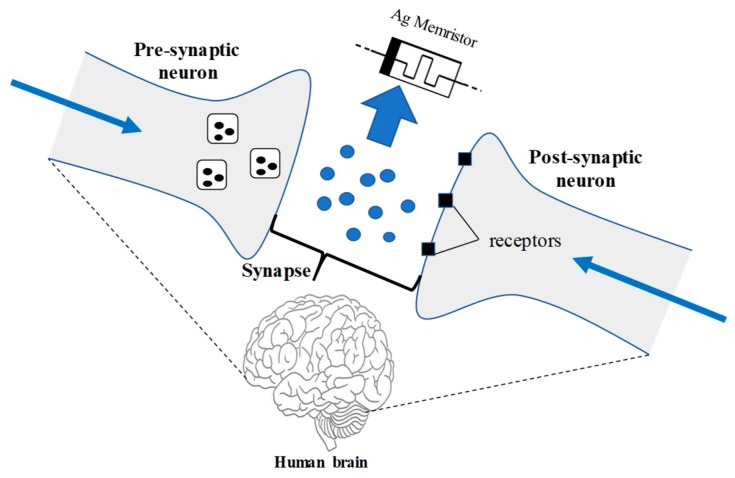
Typical block diagram of advanced neuro-electronics computing which has its synaptic emulators between the presynaptic and postsynaptic neurons.

Figure 1 shows a block diagram of advanced neuro-electronics computing, where the transmitted signal from presynaptic neurons reaches to the receptors of the postsynaptic neurons through memristor devices. The proposed memristor with Ag and Au electrodes provides an excellent neural interface approach between pre- and postsynaptic neurons, and that could bring a new paradigm in neural prostheses. Memristors are two terminal devices, it’s fundamental circuit element with metal–insulator–metal (MIM) structure, which actually maintains its resistance state once the applied switching voltage or current is removed [5,6]. However, Resistive Random-Access Memory (ReRAM), within memristor-based nanodevices, seem to fulfill the requirement of advanced neuromorphic computing [7], because they can be scaled down to the dimensions smaller than 15 nm with non-volatile, multiple-state operations and low-energy electrical switching [8,9,10,11]. A recent survey investigates various metal memristor oxides, such as ZnO, NiO and CuO among the switching materials; however, these memristor oxides are considered as materials with great importance due to their rapid speed of resistive switching [12,13,14,15]. Moreover, titanium dioxide (TiO_2_) is considered to be the most promising class of switching material [16,17] among oxides, because it is very ubiquitous among several areas such as solar cells [18,19], gas sensors [20], memristors [21,22], etc. A metal–oxide sandwich structure is promising towards the nonvolatile memory devices. The resistive switching occurs at the interface of the metal electrode and the oxide region, which follows a conductive path, also known as the interface path [23,24]. The continuous flow of oxygen vacancies into the vicinity of the interface layer reduce the digital barrier in the low resistance state. While in contrast, the oxygen vacancies are repelled away under an electric field with opposite polarity from the interface region, and also restore the electronic barrier to regain a high resistance state [24,25].

The properties of minimum power consumption, simple composition and compatible processing make memristors a great potential candidate for superior characteristics in many applications, such as novel logical devices [26] and artificial neuromorphic systems [7,8,9,10,11,12,13,14,15,16,17,18,19,20,21,22,23,24,25,26,27]. The Ag/TiO_2_ NPs/TiO_2_ Thin Film (TF)/Si-based, non-volatile memristor device for neuromorphic application has been demonstrated in our previous work [28]. In this present work, the physical vapor deposition technique is employed to fabricate two different memristor devices with top layers, i.e., the Ag and Au electrodes. Moreover, comparative studies of both proposed memristor layers show the charge storage capability under different sweeping voltage conditions towards neuromorphic computing. The organization of the paper follows as: Section 2. describes the fabrication and the characterization process of Au- and Ag-based memristor devices, while performance and evaluation with application in image classification are discussed in Section 3., and finally, our conclusion has been appended in Section 4.

## 2. Materials and Methods

**Figure 2:**
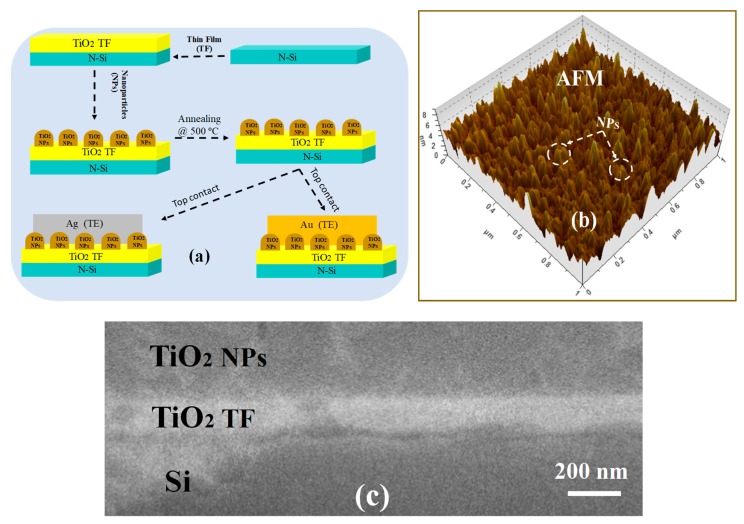
Devices with the (**a**) Schematic representation of fabrication process flow and (**b**) three-dimensional (3D) atomic force microscopy (AFM) image of the sample (**c**) Scanning electron microscopy (SEM) image of the Titanium Dioxide Thin Film (TF) (TiO_2_ TF) and Titanium Dioxide Nanoparticles (TiO_2_ NPs).

This section explores the device fabrication and its characterization with multilayer memristors that are represented as n-Si/TiO_2_ Thin Film (TF) /TiO_2_NPs/Ag and n-Si/TiO_2_TF/TiO_2_NPs/Au, respectively. The perpendicular deposition is carried out with 99.999% pure Titanium Dioxide (TiO_2_, Manufacturer: MTI USA) to deposit 40 nm TiO_2_ thin films (TF) at a base pressure of 2 × 10^−5^ mbar on n-type Si<100> substrate (~ 30 ohm-cm). Then TiO_2_ nanoparticles of 15 nm are grown over a 40 nm TiO_2_ TF by a glancing angle deposition process [29]. The deposition rates are considered as 1.2 Å s^−1^ for the growth process. The substrate holder is kept almost ≈ 18 cm in distance from the source material, where the substrate is fixed at an optimized angle of 85° and rotation of 460 rpm. In the process, the sample was annealed in the open-air condition at 500 °C for 1-hour, inside the tube furnace (GSL-1700X, MTI, Richmond, CA, USA), using the oven heating and cooling ramp at 30 °C/min. Furthermore, two different materials Au and Ag metal contact is grown on the top of developed multilayer n-Si/TiO_2_ TF/TiO_2_ NPs, respectively. The device schematic representation of the memristors layers i.e., n-Si/TiO_2_ TF/TiO_2_ NPs/Au and n-Si/TiO_2_ TF/TiO_2_ NPs/Ag are shown in Figure 2a. While the morphology of the sample: n-Si/TiO_2_ TF/TiO_2_ NPs is shown in Figure 2b, where grown nanoparticles are clearly visible in the 3D figure captured by Atomic Force Microscopy (AFM) and Figure 2c scanning electron microscopy (SEM) image of the TiO_2_ TF and TiO_2_ NPs. 

## 3. Results and Discussion

In this section, the performance and evaluation of the multilayer memristor with comparative studies are described in the following subsections.

### 3.1. Memristors Performance

**Figure 3:**
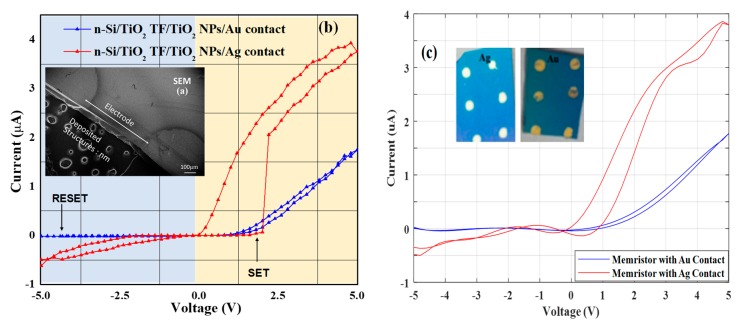
A multilayer memristor devices with its (**a**) sample morphology SEM image (**b**) I–V characteristics under different sweeping voltages and (**c**) statistical fit I–V curve.

The image of both the devices is captured by Scanning Electron Microscopy (SEM) shown in Figure 3). While the current (I) versus voltage (V) characteristics and photocurrent spectrum of the TiO_2_ NPs-based detector are measured by using a Semiconductor Parameter Analyzer (Agilent 4156B, Agilent Technologies, Inc., Santa Clara, CA, USA), which are shown in Figure 3b, and the statistical fit curve (MATLAB-2018) of I–V characteristics based on the experiments is shown in Figure 3c.

The resistive switching phenomenon observed by the I–V characteristics of Ag/TiO_2_ NPs and Au/TiO_2_ NPs devices under different sweeping voltages. Figure 3b shows the I–V curve, where the voltage applied on the top and bottom electrode with respect to ground. As the voltage is swept +Ve bias, the device exhibits an abrupt increase of current by three times near 0.2 V (called set the voltage) for Ag, whereas it is as a slight increase of current at 1 V for Au (set voltage). These indicate the transmission of the electrical resistance from a high state (‘OFF’) to a low state (‘ON’). Once the applied voltage exceeds the set and reset voltage, both devices are remaining on state at 5 V. While beyond the reset voltage, a sharp decrement in current shows a negative differential resistance (NDR) behavior for both in Au as well as in Ag. It is found that NDR is more prominent in Ag as compared to Au from the I-V characteristics. The region beyond the NDR region (>5 V) appeared to continue on a trend extending from the OFF state. The I–V characteristics are again measured and it is then found that the device exhibited an almost equivalent track of the current which is shown in the 1st loop, and indicates a rewritable memory effect. Therefore, now the device could be set from OFF to ON by applying a voltage slightly higher than the set voltage while resetting from ON to OFF by a voltage beyond the NDR region. The negative differential resistance and charge retention is more pronounced in Ag as compared to the Au device because of a subsequent fall in electrical resistance with applied voltage.

A statistical fit of the experimented data on a nonlinear polynomial equation $I=a+\mathrm{bv}+{\mathrm{cv}}^{2}+{\mathrm{dv}}^{3}+{\mathrm{ev}}^{4}+\ldots$ was performed using curve fitting techniques. The polynomial coefficient is different for dv>0 and dv<0, as the system is forming a hysteresis. The least square curve fitting with multi start approach is chosen to find the coefficient of the polynomial, while considering the abovementioned experimental results. Here two functions are calculated separately for the two cases when dv>0 and dv<0. The current output readings are taken between −5 V to 5 V for dv>0 and 5 V to −5 V for dv<0 with a 0.2 V interval [30]. 

For the Au electrode, the statistical fit functions form a hysteresis loop between −0.8 V to 4.7 V and which can be described using Equations (1) and (2) respectively.

(1) $$I=\;\left(-0.0008v^{4}+0.0588v^{3}+0.3961v^{2}+0.4648v-0.4095\right)*10^{-6}\;\mathrm{when}\;\mathrm{dv}>0$$

(2) $$I=\left(-0.0049v^{4}+0.0465v^{3}+0.4792v^{2}+0.7430v-0.2579\right)*10^{-6}\;\mathrm{when}\;\mathrm{dv}<0$$

Whereas, for Ag contact, the functions form a hysteresis loop between −2.1 V to 4.8 V which can be described using Equations (3) and (4), respectively

(3) $$I=\;\left(0.0002v^{8}+0.0010v^{7}-0.0036v^{6}-0.0234v^{5}+0.0184v^{4}+0.2128v^{3}\;+0.1703v^{2}-0.1716v-0.1061\right)*10^{-5}\;\mathrm{when}\;\mathrm{dv}>0$$

(4) $$I=\left(-0.3895v^{10}\right)*10^{-5}\;\mathrm{when}\;\mathrm{dv}<0$$

Table 1 shows the comparison of statistical fit functions with experimental data. A high correlation value and low error between the experimental and the calculated value proves the robustness of the equation. The correlation and mean square error (MSE) were calculated using Equations (5) and (6).

(5) $$Correlation=\frac{E\left[\left(ED-\mu ED\right)\left(SD-\mu SD\right)\right]}{\sigma_{ED}\sigma_{SD}}$$

(6) $$MSE=\frac{1}{n}\overset{n}{\underset{i=1}{\sum }}{\left(ED-SD\right)}^{2}$$

In Equations (5) and (6), ED stands for Experimental Data, SD for Statistical fit Data, n is the total number of datapoints, µ stands for mean and σ stands for standard deviation.

**Figure 4:**
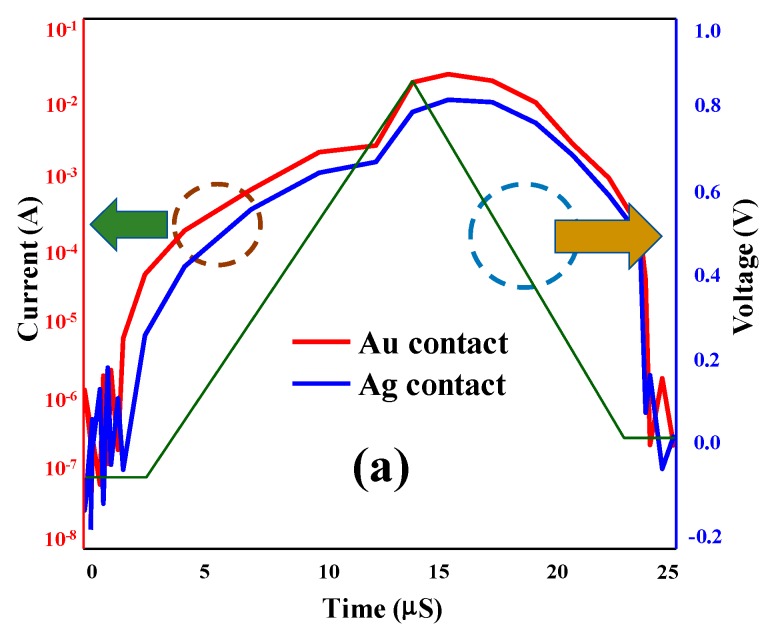

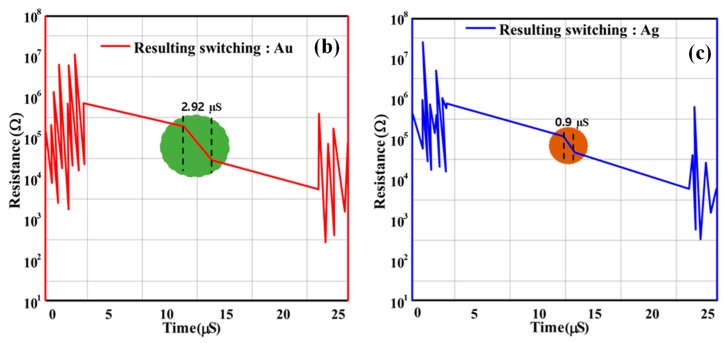
Comparative devices performances (**a**) I–V–t curve for both Au/TiO_2_ NPs and Ag/TiO_2_ NPs. (**b**) R versus switching time for Au/TiO_2_ NPs and (**c**) R versus switching time for Ag/TiO_2_ NPs.

Figure 4a shows plotting response of Current–Voltage–Time (I–V–t) curve for both Au and Ag devices, while the switching time of Au/TiO_2_ NPs- and Ag/TiO_2_ NPs-based devices are shown in Figure 4b,c, respectively. The resultant switching period is plotted for both devices by measuring the current–voltage and resistance values according to ohmic law with respect to time. The point of interaction is therefore considered as the inception-point and as end-point of switching for variation in resistance in the set process. Therefore, switching time can be easily calculated, and it is observed that the values of switching are 0.9 µS in the case of the Ag/TiO_2_ device and 2.92 µS for the Au/TiO_2_ device. Hence it is clear at the point of observation that the switching time of the Ag/TiO_2_ device is much faster than the Au/TiO_2_. This is due to the fact that conductive path for the Ag/TiO_2_-based device would be created faster and leads to provide faster operations. 

**Figure 5:**
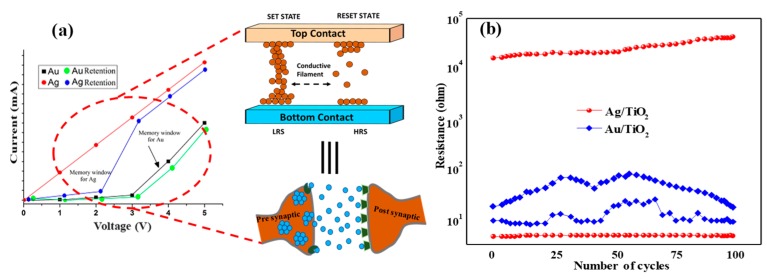
(**a**) Comparison of the I–V curve for both memristors with relation to the memory cross-bar and synaptic concept (**b**) Endurance cycle of the devices.

The comparative I–V curve of both devices is shown in Figure 5a, where a memory window of endurance testing for Ag is more pronounced than for Au. The memory windows of 1.6 V for Ag/TiO_2_ and 0.8 V for Au/TiO_2_ are observed in the retention of the I–V curve. Figure 5a also reveals the interfacing of memory devices with a brain-inspired computing application. Furthermost, the endurance properties of both of the devices shown in Figure 5b where the Ag/TiO_2_ devices present the stable endurance of 100 cycles because of greater oxygen vacancies and the strong conductive path beneath the device. The crossbar memory pattern is very essential towards the artificial conjugation of presynaptic and postsynaptic neurons. Here it is perceived that the Ag-based electrodes memristor system can be very useful for mimicking the brain function.

### 3.2. Capacitance Vs Voltage

**Figure 6:**
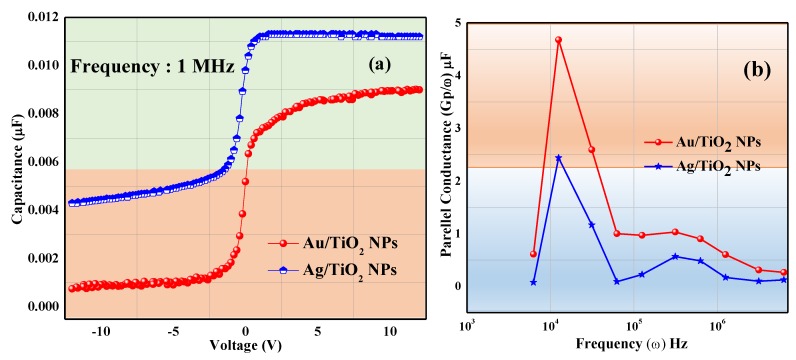
(**a**) C–V characteristics for the Ag/TiO_2_ NPs and Au/TiO_2_ NPs memristors under different sweeping voltages at 1 MHz (**b**) *G*p*/ω* Vs *ω* at 0 V.

The capacitance Vs voltage (C–V) graph at 1 MHz frequency is shown in Figure 6a. It is feasible from the graph that the Ag/TiO_2_ NPs device is higher (0.014 µF) as compared to Au/TiO_2_ NPs (0.007 µF) at 0V capacitance. It is noticed that the width of the depletion region decreases with the increment of applied voltage, hence the capacitance gets increased. It is due to the low interface traps in the Au/TiO_2_ NPs device that reduces electron mobility, receiving low mem-conductance [31]. The parallel conductance over angular frequency (*G*p*/ω*) Vs frequency (*ω*) plot (Figure 6b) reveals that the device with the Ag/TiO_2_ (2.45 µF) combination shows low mem-interface density, as compared to the Au/TiO_2_ NPs (4.69 µF), which may be because of the fact that the series resistance is ignored in the process. The Hill–Coleman methodology is considered to find the memristive traps density $D_{\mathrm{it}}$ for the single frequency method from the conductance curve which can be described in Equations 5 and 6, respectively [32].

(7) $$D_{\mathrm{it}}=\frac{1}{q}\left(\frac{C_{\mathrm{OX}}C_{\mathrm{LF}}}{C_{\mathrm{OX}}-C_{\mathrm{LF}}}-\frac{C_{\mathrm{OX}}C_{\mathrm{HF}}}{C_{\mathrm{OX}}-C_{\mathrm{HF}}}\right)$$

(8) $$D_{\mathrm{it}}=\;\frac{\left(\frac{2}{\mathrm{qA}}\right)\left(\frac{G_{m\_\max}}{\omega }\right)}{{\left(\frac{G_{m\_\max}}{{\omega C}_{\mathrm{ox}}}\right)}^{2}+{\left(1-\frac{C_{m}}{C_{\mathrm{ox}}}\right)}^{2}}$$

Here, from the above Equation 6, the interface trap density $D_{\mathrm{it}}$ for Ag/TiO_2_ NPs is calculated as 3.68 × 10^10^ eV^−1^cm^−2^, which is quite a deal smaller than the Au/TiO_2_ NPs (whereas 6.45 × 10^12^ eV^−1^cm^−2^). It may be due to the decrease in the surface to volume ratio in between Au and the insulating oxide. Therefore, the Ag/TiO_2_ NPs device is more suitable and has better storage for advanced neuro-computing applications.

### 3.3. Application with Ag Based Memristor for Image Classification.

From the above evaluation, it is found that the Ag memristor shows excellent performance in terms of charge storage and switching characteristics as compared to the Au memristor. So, the Ag contact-based memristor can be a good candidate for developing artificial synaptic emulators for neuromorphic systems. 

**Figure 7:**
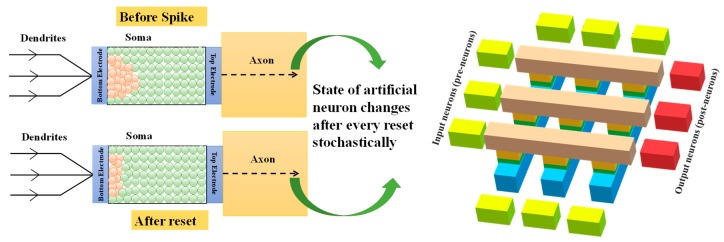
Typical functionality of the actual neuron and pre- and post-neuron crossbar structure.

In order to validate a faithful clarification of the proposed application, this work established an Ag/TiO_2_ NPs/ TiO_2_ TF/Si-based memristor crossbar structure to implement a spike neuron network towards 2D image processing solutions. Figure 7 shows the typical functionality of the pre- and post-neuron crossbar structure with the base of an actual neuron.

There are three major aspects of the artificial neurons, namely the dendrites (input), soma (body) and axon (output). As a processor, the soma does the work, receiving inputs via the dendrites, then generating the output at the axon. Use synaptic weights, the artificial neurons obtain the potential at the dendrites and modulate it, leading to changes in the physical state that gradually increase the electrical conductance. When multiple input arrives, this results in a sequential rise in electrical conductance. The overall conductance will increase until the threshold point is achieved. Thus, the neuron fires the spike, after which it can reset to a nonconducting state. The artificial neuron is in a slightly different state, before the pulse is arrived, and thus provides the inherent stochasticity or randomness to the functioning of the artificial neuron.

**Figure 8:**
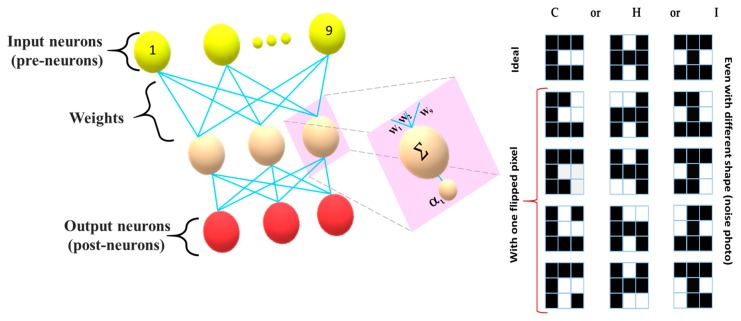
Three layers of neural network and the set of used patterns.

The base of actual neuron could be considered as a 3 × 3 crossbar memristor for a spiking neuron configuration. The pre-neurons (yellow color) and post-neurons (red color) are connected through the memristor crossbar array, as shown in Figure 8. The system consists of nine (9) inputs (i) and three (3) outputs, which are fully connected to the 5 × 3 = 15 synaptic weight (*W*i), where nine (9) inputs represent the pixel values. The system is checked on the range of N = 15 patterns, with three conventional letters (‘C’, ‘H’ and ‘I’), including the three sets of four noisy types letter variants; it is created by flipping any one of the pixels of the ideal image. The modified integrate and fire (LIF) model is employed to emulate the neuronal characteristics. Thus, the input neuron will fire a spike voltage which represents the white and black pixel. Here each synapse corresponds to one memristor in the cross bar. The memristor will produce different weights represented by its conductance value depending on the input spike voltage. In the process the crossbar array will activate one single neuron (*α*_i_) (either of C or H or I) from the output layer, depending upon the integrated synaptic weight (Σ). This will eventually increase its membrane potential gradually, and if the charge accumulations of the neuron exceeds, this is to achieve a predefined threshold.

**Figure 9:**
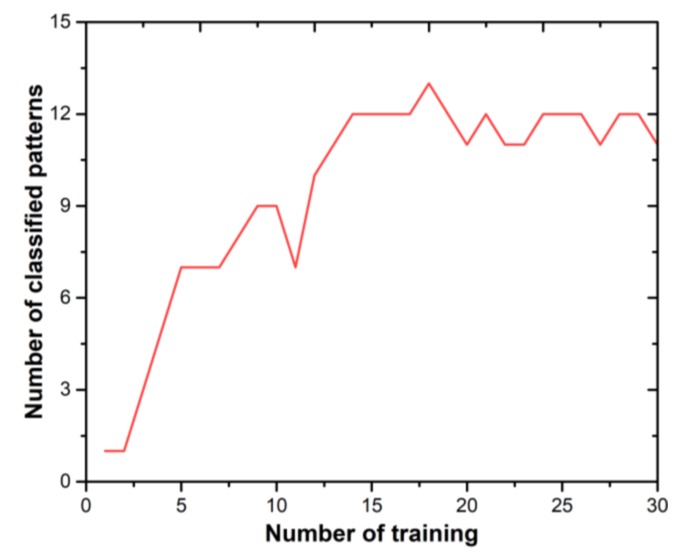
Experimental result of the pattern classification.

The training and testing image are same due to a very limited size. The assumed findings have successfully classified the image on average after 18 training iterations (shown in Figure 9.)

## 4. Conclusions

This work presents the comparative study of memristor devices with TiO_2_ NPs/Ag and TiO_2_ NPs/Au electrodes for synaptic emulators in an advanced neurocomputing application. The sample was characterized by AFM and SEM equipment. The Memristive electric switching characteristics under different sweeping voltages lead into the low resistance and high resistance states where electron movement and oxygen vacancies are found to be maximum for the device with Ag contact. The large memory window (1.6 V) and good retention characteristics were observed for the Ag/TiO_2_ device. The Resistance Vs Time graph revealed the maximum switching speed and stability of the Ag/TiO_2_ device over that of Au/TiO_2_. The higher capacitance and lower *G*p*/ω* make the Ag/TiO_2_ NPs device more prominent for low power applications. The application of the Ag-based memristor crossbar pattern shows the perfect demonstrations of the spike neuron network for the 2D image processing task. Finally, we can conclude that the Ag contact-based memristor pattern may consume low cost, less power and less complexity, which is in-fact very valuable towards the study and development of neuro–bio–morphic systems.

## Figures and Tables

**Table 1:** Comparison of statistical fit functions with experimental data.

Device	Correlation Value between Experimental Data and Statistical Fit Data	Mean Square Error between Experimental Data and Statistical Fit Data
**Memristor with Au Contact**	0.9957	2.228 × 10^−13^
**Memristor with Ag Contact**	0.9952	2.147 × 10^−12^
